# Supplementary material for: A Likelihood Approach for Real-Time Calibration of Stochastic Compartmental Epidemic Models
Source: PLoS Comput Biol. 2017 Jan 17;13(1):e1005257. doi: 10.1371/journal.pcbi.1005257 (PMC5240920; doi:10.1371/journal.pcbi.1005257)
Supplement: S1 File — (TAR.GZ) [file pcbi.1005257.s014.tar.gz › HSPH_Online-SI-Revision/EnKF07/n100-severe/n100-severe_EnKF2_table4.pdf]

1	newly 3 weeks spec	1675.	1599.9	{132., 3809.}	75.1248	0.457388	newly 3 weeks cumul	7166.	20 905.8	{5105., 57 331.}	205.58	0.356437	newly 5 weeks spec	875.	240.29	{8., 623.}	74.7966	1.05244	newly 5 weeks cumul	9240.	21 735.8	{5599., 58 000.}	154.317	0.307465	newly 46 weeks cumul	11 305.	22 041.6	{5605., 58 327.}	122.724	0.286398
2	newly 3 weeks spec	260.	965.29	{34., 1217.}	326.573	0.519415	newly 3 weeks cumul	1782.	5826.51	{243., 10 452.}	277.713	0.521542	newly 5 weeks spec	65.	333.02	{4., 335.}	481.323	0.626386	newly 5 weeks cumul	1949.	6731.42	{267., 10 556.}	295.321	0.524044	newly 53 weeks cumul	2047.	7207.72	{277., 10 559.}	302.129	0.527653
3	newly 3 weeks spec	220.	3312.33	{181., 9909.}	1418.44	0.768901	newly 3 weeks cumul	1526.	11 983.2	{968., 26 789.}	703.405	0.637243	newly 5 weeks spec	41.	1768.02	{55., 6853.}	4219.56	1.07568	newly 5 weeks cumul	1670.	16 225.	{1257., 42 227.}	886.831	0.682253	newly 54 weeks cumul	1741.	20 617.6	{1445., 85 183.}	1097.3	0.73658
4	newly 3 weeks spec	1102.	1201.4	{148., 2017.}	122.793	0.549142	newly 3 weeks cumul	5564.	6861.95	{1259., 13 498.}	123.522	0.445355	newly 5 weeks spec	463.	399.73	{17., 389.}	116.093	0.731763	newly 5 weeks cumul	6701.	7949.76	{1493., 16 042.}	122.507	0.465595	newly 52 weeks cumul	7579.	8594.96	{1498., 20 617.}	120.747	0.486218
5	newly 3 weeks spec	2014.	1163.	{91., 1937.}	80.7051	0.71876	newly 3 weeks cumul	7895.	18 110.3	{6076., 50 009.}	143.883	0.254125	newly 5 weeks spec	1193.	202.58	{2., 788.}	84.0386	1.62506	newly 5 weeks cumul	10 658.	18 761.5	{6085., 50 142.}	104.746	0.229468	newly 46 weeks cumul	14 294.	19 002.2	{6085., 50 144.}	91.9569	0.295133
6	newly 3 weeks spec	1306.	1396.39	{137., 1298.}	124.417	0.581437	newly 3 weeks cumul	6189.	6880.61	{1852., 7620.}	109.657	0.439274	newly 5 weeks spec	562.	480.44	{12., 666.}	116.231	0.781381	newly 5 weeks cumul	7603.	8190.87	{2031., 10 227.}	111.267	0.467495	newly 52 weeks cumul	8616.	8882.1	{2202., 13 600.}	109.816	0.48839
7	newly 3 weeks spec	678.	581.33	{36., 1481.}	87.8038	0.597452	newly 3 weeks cumul	3914.	7690.84	{644., 12 906.}	160.622	0.398655	newly 5 weeks spec	239.	154.7	{1., 264.}	90.6109	1.03311	newly 5 weeks cumul	4550.	8126.1	{670., 13 635.}	147.968	0.412336	newly 53 weeks cumul	4820.	8364.3	{673., 13 753.}	145.005	0.41973
8	newly 3 weeks spec	1197.	737.3	{3., 2530.}	115.166	1.42457	newly 3 weeks cumul	5691.	6481.69	{852., 8827.}	128.696	0.56033	newly 5 weeks spec	560.	269.63	{0., 1230.}	117.595	2.35826	newly 5 weeks cumul	7047.	7163.82	{929., 13 839.}	125.674	0.629998	newly 49 weeks cumul	8398.	7907.99	{929., 22 843.}	125.72	0.698274
9	newly 3 weeks spec	121.	4006.82	{0., 19 733.}	3256.36	1.60441	newly 3 weeks cumul	1328.	22 869.8	{2036., 80 487.}	1629.43	0.923197	newly 5 weeks spec	4.	2347.15	{0., 10 405.}	58 622.8	2.2301	newly 5 weeks cumul	1373.	28 326.6	{3001., 87 755.}	1967.43	1.00197	newly 54 weeks cumul	1373.	37 079.3	{5079., 102 243.}	2603.6	1.14782
10	newly 3 weeks spec	840.	3407.03	{276., 8812.}	327.377	0.482099	newly 3 weeks cumul	4139.	35 193.9	{5123., 97 260.}	753.624	0.70941	newly 5 weeks spec	240.	398.08	{10., 1285.}	143.283	0.634609	newly 5 weeks cumul	4837.	36 713.7	{5437., 99 455.}	662.838	0.675832	newly 47 weeks cumul	5228.	37 035.4	{5447., 99 577.}	612.931	0.655141
11	newly 3 weeks spec	467.	620.96	{29., 1868.}	107.482	0.553554	newly 3 weeks cumul	2925.	8336.41	{338., 11 412.}	229.965	0.447019	newly 5 weeks spec	130.	165.91	{1., 295.}	124.438	0.871564	newly 5 weeks cumul	3288.	8803.56	{358., 15 924.}	213.859	0.445133	newly 53 weeks cumul	3458.	9047.68	{360., 19 925.}	208.645	0.447112
12	newly 3 weeks spec	1748.	988.59	{74., 3210.}	88.6081	0.865807	newly 3 weeks cumul	7573.	16 239.4	{2881., 55 213.}	149.44	0.339419	newly 5 weeks spec	887.	227.9	{1., 896.}	94.221	1.79167	newly 5 weeks cumul	9747.	16 901.1	{3925., 55 345.}	124.906	0.354232	newly 46 weeks cumul	12 152.	17 380.	{5597., 55 347.}	109.784	0.378577
13	newly 3 weeks spec	1801.	1749.63	{260., 3573.}	91.5386	0.551085	newly 3 weeks cumul	7188.	22 141.9	{7851., 68 492.}	215.785	0.352083	newly 5 weeks spec	954.	294.2	{10., 452.}	92.9119	1.21192	newly 5 weeks cumul	9478.	23 128.6	{7924., 69 110.}	155.928	0.277636	newly 46 weeks cumul	11 854.	23 409.3	{7954., 70 441.}	122.686	0.258773
14	newly 3 weeks spec	2332.	1429.19	{146., 3361.}	69.4876	0.605205	newly 3 weeks cumul	8946.	19 547.6	{5969., 54 223.}	132.214	0.281754	newly 5 weeks spec	1422.	226.86	{4., 542.}	88.8565	1.40296	newly 5 weeks cumul	12 296.	20 322.5	{5976., 56 214.}	91.7061	0.24138	newly 46 weeks cumul	16 703.	20 524.7	{5976., 56 219.}	75.0794	0.276407
15	newly 3 weeks spec	957.	1517.72	{206., 2815.}	121.996	0.473987	newly 3 weeks cumul	4781.	8211.42	{653., 10 839.}	136.23	0.46512	newly 5 weeks spec	410.	616.3	{39., 1176.}	109.79	0.509809	newly 5 weeks cumul	5823.	9778.19	{949., 15 246.}	130.18	0.452557	newly 48 weeks cumul	6577.	11 618.5	{1661., 22 868.}	126.195	0.416578
16	newly 3 weeks spec	2249.	3040.52	{1., 9247.}	131.32	0.996534	newly 3 weeks cumul	9354.	34 179.7	{6181., 93 014.}	279.423	0.420547	newly 5 weeks spec	1205.	283.39	{0., 565.}	86.1303	1.58661	newly 5 weeks cumul	12 257.	35 412.1	{6334., 95 430.}	209.935	0.366323	newly 47 weeks cumul	15 533.	35 665.8	{6385., 95 521.}	162.854	0.346454
17	newly 3 weeks spec	539.	902.38	{20., 1957.}	150.56	0.625482	newly 3 weeks cumul	2954.	9929.82	{151., 16 934.}	286.012	0.502699	newly 5 weeks spec	198.	400.86	{1., 308.}	214.818	1.0125	newly 5 weeks cumul	3479.	10 889.2	{178., 20 988.}	265.292	0.507068	newly 53 weeks cumul	3686.	11 905.2	{186., 35 695.}	276.443	0.520471
18	newly 3 weeks spec	789.	2507.66	{85., 5570.}	291.354	0.540036	newly 3 weeks cumul	4078.	10 682.1	{701., 25 185.}	220.577	0.43438	newly 5 weeks spec	352.	1297.84	{21., 3713.}	371.341	0.753934	newly 5 weeks cumul	4968.	13 727.	{723., 33 491.}	237.37	0.462753	newly 51 weeks cumul	5544.	17 379.8	{760., 77 680.}	277.298	0.504869
19	newly 3 weeks spec	457.	2075.1	{32., 4359.}	440.197	0.679696	newly 3 weeks cumul	2528.	8884.26	{309., 17 892.}	330.379	0.568444	newly 5 weeks spec	174.	789.22	{5., 1877.}	455.586	0.887223	newly 5 weeks cumul	2969.	10 950.3	{347., 20 976.}	345.386	0.576152	newly 51 weeks cumul	3262.	13 840.	{352., 39 799.}	399.676	0.639716
20	newly 3 weeks spec	674.	1685.88	{80., 4116.}	210.009	0.524966	newly 3 weeks cumul	3591.	7286.77	{452., 12 668.}	150.585	0.429853	newly 5 weeks spec	266.	920.59	{16., 4052.}	316.071	0.687273	newly 5 weeks cumul	4252.	9447.1	{574., 21 800.}	170.864	0.453255	newly 49 weeks cumul	4608.	12 614.5	{664., 43 946.}	222.412	0.494816
21	newly 3 weeks spec	521.	616.1	{35., 1686.}	101.731	0.554019	newly 3 weeks cumul	3210.	8028.28	{568., 10 204.}	197.322	0.395415	newly 5 weeks spec	157.	162.26	{0., 327.}	106.688	0.963684	newly 5 weeks cumul	3675.	8486.32	{606., 12 531.}	181.909	0.39448	newly 53 weeks cumul	3774.	8738.22	{613., 15 275.}	182.47	0.397035
22	newly 3 weeks spec	558.	1947.87	{121., 4445.}	319.83	0.505976	newly 3 weeks cumul	3543.	6602.66	{755., 9431.}	164.167	0.430813	newly 5 weeks spec	178.	1419.	{16., 4383.}	760.596	0.633963	newly 5 weeks cumul	4026.	9692.96	{930., 25 991.}	214.178	0.4602	newly 51 weeks cumul	4274.	15 667.5	{1055., 57 775.}	337.08	0.535149
23	newly 3 weeks spec	35.	1636.24	{0., 1400.}	4664.17	1.29619	newly 3 weeks cumul	468.	15 134.4	{1324., 67 323.}	3134.96	1.07538	newly 5 weeks spec	5.	838.84	{0., 726.}	16 808.4	1.79459	newly 5 weeks cumul	490.	17 126.7	{1616., 77 326.}	3396.41	1.09734	newly 55 weeks cumul	497.	19 946.	{1833., 80 995.}	3914.44	1.14005
24	newly 3 weeks spec	330.	629.17	{64., 839.}	134.518	0.384617	newly 3 weeks cumul	2438.	5053.76	{1596., 5280.}	122.724	0.264573	newly 5 weeks spec	72.	166.14	{2., 308.}	196.056	0.704973	newly 5 weeks cumul	2664.	5537.39	{1599., 5527.}	123.672	0.266551	newly 56 weeks cumul	2695.	5774.22	{1717., 5657.}	129.881	0.277545
25	newly 3 weeks spec	192.	795.88	{71., 1111.}	345.021	0.525723	newly 3 weeks cumul	1247.	5435.73	{1658., 6896.}	338.086	0.494288	newly 5 weeks spec	31.	247.76	{2., 441.}	742.839	0.841137	newly 5 weeks cumul	1365.	6122.72	{1886., 7382.}	350.554	0.498246	newly 56 weeks cumul	1367.	6558.88	{1963., 7499.}	381.684	0.523395
26	newly 3 weeks spec	830.	2411.44	{34., 8182.}	279.627	0.63604	newly 3 weeks cumul	4614.	8642.55	{475., 23 713.}	181.32	0.58572	newly 5 weeks spec	289.	977.85	{2., 2832.}	315.671	0.778026	newly 5 weeks cumul	5390.	11 165.	{621., 39 439.}	196.328	0.570606	newly 50 weeks cumul	5805.	14 330.8	{621., 69 736.}	221.931	0.53417
27	newly 3 weeks spec	578.	2090.86	{30., 2559.}	355.007	0.689936	newly 3 weeks cumul	3242.	10 541.7	{248., 9625.}	300.589	0.549819	newly 5 weeks spec	170.	628.08	{2., 1999.}	368.447	0.893269	newly 5 weeks cumul	3757.	12 297.4	{274., 13 648.}	303.481	0.561566	newly 51 weeks cumul	4031.	13 636.7	{320., 20 589.}	315.3	0.582729
28	newly 3 weeks spec	1075.	1684.29	{87., 2188.}	144.241	0.5209	newly 3 weeks cumul	4677.	6164.23	{531., 7299.}	117.065	0.468619	newly 5 weeks spec	430.	976.54	{37., 3311.}	207.321	0.610825	newly 5 weeks cumul	5845.	8383.14	{674., 11 473.}	126.592	0.481629	newly 50 weeks cumul	7034.	14 620.4	{719., 47 933.}	185.841	0.546255
29	newly 3 weeks spec	909.	1765.03	{83., 5089.}	203.114	0.643434	newly 3 weeks cumul	4464.	5556.59	{447., 16 703.}	120.757	0.556191	newly 5 weeks spec	364.	1632.57	{21., 6112.}	460.107	0.764538	newly 5 weeks cumul	5336.	9030.09	{610., 36 699.}	164.74	0.56892	newly 51 weeks cumul	5817.	13 012.9	{823., 49 407.}	211.37	0.556204
30	newly 3 weeks spec	1036.	3118.14	{432., 8298.}	230.295	0.487936	newly 3 weeks cumul	4857.	33 003.3	{4078., 98 723.}	589.101	0.620751	newly 5 weeks spec	473.	322.16	{3., 700.}	57.1586	0.663973	newly 5 weeks cumul	6087.	34 267.2	{4366., 100 677.}	474.289	0.566495	newly 47 weeks cumul	7069.	34 493.2	{4477., 100 756.}	400.671	0.521435
31	newly 3 weeks spec	35.	430.51	{35., 658.}	1135.34	0.936269	newly 3 weeks cumul	499.	3366.55	{1336., 5200.}	576.635	0.772934	newly 5 weeks spec	17.	107.71	{1., 208.}	564.529	0.784535	newly 5 weeks cumul	538.	3686.54	{1340., 5368.}	587.193	0.771176	newly 56 weeks cumul	540.	3810.96	{1340., 5378.}	607.696	0.780257
32	newly 3 weeks spec	307.	3228.13	{373., 9750.}	959.554	0.668128	newly 3 weeks cumul	2113.	18 786.	{6684., 56 667.}	792.188	0.779782	newly 5 weeks spec	67.	496.96	{26., 1934.}	669.463	0.633746	newly 5 weeks cumul	2352.	20 582.	{6804., 64 352.}	778.215	0.761758	newly 54 weeks cumul	2475.	20 902.3	{6820., 65 372.}	747.685	0.745307
33	newly 3 weeks spec	386.	1724.46	{171., 2219.}	386.482	0.455409	newly 3 weeks cumul	2219.	11 434.4	{4297., 11 572.}	420.888	0.523797	newly 5 weeks spec	106.	382.55	{6., 698.}	351.858	0.736286	newly 5 weeks cumul	2533.	12 653.1	{4405., 13 591.}	405.287	0.500089	newly 54 weeks cumul	2695.	13 188.5	{4409., 16 374.}	395.222	0.49261
34	newly 3 weeks spec	1133.	2294.72	{0., 6102.}	188.913	0.983564	newly 3 weeks cumul	5334.	24 285.6	{7701., 54 771.}	358.01	0.538938	newly 5 weeks spec	504.	490.	{0., 1861.}	139.087	1.6294	newly 5 weeks cumul	6608.	25 831.8	{7706., 54 902.}	293.918	0.47149	newly 46 weeks cumul	7621.	26 281.1	{7706., 54 903.}	248.287	0.419052
35	newly 3 weeks spec	193.	1253.07	{10., 2950.}	632.585	0.805798	newly 3 weeks cumul	1207.	13 439.8	{2413., 26 701.}	1016.42	0.728457	newly 5 weeks spec	21.	370.62	{0., 722.}	1784.95	1.63419	newly 5 weeks cumul	1310.	14 381.4	{2433., 33 080.}	1000.98	0.735756	newly 55 weeks cumul	1313.	16 448.3	{2864., 73 612.}	1155.9	0.786201
36	newly 3 weeks spec	618.	492.3	{32., 1270.}	88.589	0.66252	newly 3 weeks cumul	3733.	7548.16	{448., 9845.}	169.329	0.44279	newly 5 weeks spec	195.	117.98	{0., 285.}	93.0462	1.13404	newly 5 weeks cumul	4250.	7886.09	{543., 12 533.}	156.448	0.451026	newly 53 weeks cumul	4446.	8059.04	{592., 15 067.}	153.801	0.457772
37	newly 3 weeks spec	114.	1016.81	{3., 1451.}	863.184	0.82902	newly 3 weeks cumul	920.	12 739.1	{2161., 27 320.}	1288.23	0.812887	newly 5 weeks spec	26.	398.93	{0., 564.}	1567.73	1.78972	newly 5 weeks cumul	1039.	13 710.1	{2687., 33 898.}	1223.5	0.797379	newly 55 weeks cumul	1048.	14 916.8	{3278., 39 189.}	1327.38	0.832067
38	newly 3 weeks spec	477.	1208.04	{104., 2831.}	207.367	0.444472	newly 3 weeks cumul	2807.	12 370.5	{727., 50 440.}	372.699	0.481341	newly 5 weeks spec	136.	336.3	{7., 295.}	230.044	0.574796	newly 5 weeks cumul	3197.	13 296.1	{774., 55 472.}	349.626	0.477486	newly 53 weeks cumul	3359.	13 654.7	{859., 55 490.}	341.294	0.475349
39	newly 3 weeks spec	862.	2042.07	{83., 4909.}	217.572	0.52868	newly 3 weeks cumul	4078.	7630.56	{439., 12 004.}	170.988	0.500572	newly 5 weeks spec	352.	908.87	{32., 2402.}	236.963	0.612971	newly 5 weeks cumul	4972.	9874.06	{528., 26 205.}	177.89	0.498419	newly 50 weeks cumul	5595.	13 576.2	{589., 38 537.}	216.881	0.534433
40	newly 3 weeks spec	607.	694.85	{57., 1050.}	102.822	0.488288	newly 3 weeks cumul	3292.	6292.3	{443., 11 271.}	152.798	0.411211	newly 5 weeks spec	210.	372.76	{8., 290.}	190.962	0.723952	newly 5 weeks cumul	3890.	7131.7	{496., 11 570.}	147.176	0.421139	newly 53 weeks cumul	4289.	8612.41	{507., 13 632.}	167.016	0.443457
41	newly 3 weeks spec	231.	1142.66	{2., 2938.}	495.065	1.0308	newly 3 weeks cumul	1656.	11 538.6	{2355., 14 659.}	601.918	0.567877	newly 5 weeks spec	65.	842.26	{0., 1718.}	1332.62	2.07842	newly 5 weeks cumul	1848.	13 295.	{2370., 23 715.}	625.438	0.578811	newly 55 weeks cumul	1919.	18 784.3	{2371., 82 906.}	885.166	0.637564
42	newly 3 weeks spec	1150.	2099.77	{232., 4037.}	125.643	0.406903	newly 3 weeks cumul	5514.	10 590.5	{1059., 16 717.}	136.476	0.396227	newly 5 weeks spec	480.	930.85	{75., 2431.}	134.481	0.442442	newly 5 weeks cumul	6798.	12 898.1	{1287., 23 645.}	131.805	0.388021	newly 48 weeks cumul	7662.	15 636.4	{1543., 33 212.}	138.468	0.383241
43	newly 3 weeks spec	1025.	2561.02	{111., 7019.}	210.89	0.582793	newly 3 weeks cumul	4521.	29 019.8	{3330., 103 412.}	551.585	0.565022	newly 5 weeks spec	563.	289.19	{3., 740.}	72.1687	0.987303	newly 5 weeks cumul	5852.	30 144.1	{3523., 105 188.}	429.626	0.509712	newly 47 weeks cumul	6825.	30 336.8	{3588., 105 339.}	362.239	0.473172
44	newly 3 weeks spec	487.	3206.87	{0., 8885.}	595.764	1.14784	newly 3 weeks cumul	3260.	17 097.6	{3747., 42 612.}	431.209	0.596283	newly 5 weeks spec	138.	1116.88	{0., 1961.}	749.536	1.0882	newly 5 weeks cumul	3654.	20 133.3	{4372., 52 210.}	457.103	0.599326	newly 54 weeks cumul	3820.	21 871.8	{4915., 53 594.}	477.208	0.608044
45	newly 3 weeks spec	960.	1370.14	{185., 1186.}	130.635	0.390489	newly 3 weeks cumul	5111.	7043.75	{2207., 8991.}	112.426	0.306323	newly 5 weeks spec	424.	462.27	{18., 547.}	120.002	0.583579	newly 5 weeks cumul	6116.	8309.49	{2281., 11 877.}	114.353	0.322734	newly 52 weeks cumul	6740.	8958.27	{2285., 15 017.}	114.081	0.33568
46	newly 3 weeks spec	833.	2137.84	{50., 7311.}	278.816	0.785968	newly 3 weeks cumul	4606.	6622.19	{402., 28 758.}	146.097	0.594374	newly 5 weeks spec	254.	1492.33	{3., 1048.}	614.398	1.00194	newly 5 weeks cumul	5399.	10 057.6	{461., 44 806.}	191.099	0.625481	newly 51 weeks cumul	5711.	12 698.2	{470., 46 166.}	227.518	0.637379
47	newly 3 weeks spec	1599.	2416.19	{192., 6537.}	113.129	0.45735	newly 3 weeks cumul	6760.	38 445.7	{14 568., 89 999.}	468.723	0.65145	newly 5 weeks spec	834.	142.8	{3., 465.}	82.8777	1.2916	newly 5 weeks cumul	8781.	39 167.2	{14 849., 92 844.}	346.045	0.546109	newly 47 weeks cumul	10 888.	39 222.	{14 850., 92 982.}	260.232	0.453564
48	newly 3 weeks spec	1207.	1450.3	{68., 1942.}	136.486	0.651343	newly 3 weeks cumul	5943.	6871.04	{459., 7956.}	117.476	0.514777	newly 5 weeks spec	486.	528.53	{9., 938.}	137.08	0.853374	newly 5 weeks cumul	7196.	8283.37	{534., 11 141.}	120.726	0.541604	newly 52 weeks cumul	7925.	9107.21	{555., 15 496.}	122.589	0.560368
49	newly 3 weeks spec	149.	614.22	{74., 897.}	334.94	0.536967	newly 3 weeks cumul	1122.	4486.64	{2351., 5546.}	301.784	0.537367	newly 5 weeks spec	19.	183.37	{2., 280.}	902.053	0.879019	newly 5 weeks cumul	1199.	4999.7	{2372., 5858.}	318.847	0.543969	newly 56 weeks cumul	1212.	5268.15	{2373., 6332.}	336.425	0.552672
50	newly 3 weeks spec	163.	1061.83	{1., 3077.}	633.515	1.00599	newly 3 weeks cumul	1147.	13 370.6	{1556., 54 735.}	1066.79	0.703077	newly 5 weeks spec	19.	396.04	{0., 589.}	2099.47	1.688	newly 5 weeks cumul	1229.	14 284.4	{1942., 54 873.}	1063.44	0.719133	newly 55 weeks cumul	1262.	16 981.	{3060., 82 198.}	1246.9	0.769658
